# Intracellular Retinoid-Binding Proteins (CRBP, CRALBP, CRABP) as Emerging Drug Targets in Retinal Disease

**DOI:** 10.3390/ijms27167096

**Published:** 2026-08-07

**Authors:** Laxmi Regmi Bagale, Hye Jin Kim

**Affiliations:** College of Pharmacy, Keimyung University, 1095 Dalgubeol-daero, Dalseo-gu, Daegu 42601, Republic of Korea; laxmi809@kmu.kr

**Keywords:** intracellular lipid-binding protein, CRALBP, CRBP1, CRABP, retinal degeneration

## Abstract

Intracellular retinoid-binding proteins, including cellular retinol-binding proteins (CRBPs) and cellular retinoic acid-binding proteins (CRABPs), belong to the intracellular lipid-binding protein (iLBP) family, whereas cellular retinaldehyde-binding protein (CRALBP) is a structurally distinct retinoid-binding protein belonging to the CRAL-TRIO protein family and functions as an active regulator of retinoid trafficking, metabolism, and signaling. Although these proteins are directly implicated in inherited retinal dystrophies, retinoid-dependent cancers, and neurodegenerative diseases, they have received comparatively little attention as pharmacological targets relative to the extracellular carrier Retinol-Binding Protein 4 (RBP4). High-resolution structural studies, including atomic-resolution X-ray co-crystal structures of protein–ligand complexes, have defined the binding pocket architecture of each protein and established a basis for structure-guided drug discovery. This review critically evaluates the druggability of CRBP, CRALBP, and CRABP by integrating structural, biochemical, and pharmacological evidence. We discuss known small-molecule modulators, including the first-in-class CRBP1 inhibitor abn-CBD and next-generation non-retinoid scaffolds, alongside gene therapy strategies targeting CRALBP deficiency. We further address the selectivity challenges inherent to the conserved iLBP fold and identify future directions for the development of isoform-selective therapeutics for retinal degeneration, oncology, and neurodegeneration.

## 1. Introduction

The initial stage of vision begins with light-induced activation of visual pigments [1], which are specifically expressed in retinal photoreceptor cells and consist of an opsin apoprotein covalently linked to the chromophore 11-*cis*-retinal (11cRAL) [2,3]. Upon absorption of a photon, the chromophore 11-*cis*-retinal undergoes photoisomerization to all-*trans*-retinal, thereby activating visual pigments and initiating the phototransduction cascade [4]; subsequently, all-*trans*-retinal must be converted back to 11-*cis*-retinal to regenerate opsins and restore their light sensitivity [5]. In vertebrates, this regeneration occurs through a series of enzymatic reactions collectively known as the visual cycle, an eye-specific metabolic pathway essential for sustained light perception and the maintenance of photoreceptor cells [6]. Although chromophore regeneration is classically attributed to the RPE, cones exhibit substantially faster pigment regeneration kinetics than can be supported by the canonical RPE-dependent visual cycle alone. This rapid regeneration is additionally facilitated by an intraretinal “cone” visual cycle that is primarily mediated by Müller glial cells [7,8,9]. In the Müller cell-mediated cone visual cycle, all-trans-retinol generated from bleached cones is taken up by Müller glial cells, where it undergoes dihydroceramide desaturase-1 (DES1) dependent isomerization to 11-cis-retinol. The newly formed 11-cis-retinol is selectively bound and stabilized by CRALBP and transported back to cones, where cone-specific retinol dehydrogenase activity, including RDH10, catalyzes its oxidation to 11-cis-retinal for rapid visual pigment regeneration [10,11]. The contribution of the Müller cell-mediated visual cycle to cone pigment regeneration is linked to the high metabolic demand of cones and their requirement for rapid chromophore replenishment beyond that provided by the canonical RPE-dependent pathway alone. The photoreceptor–RPE–Müller cell network functions as an integrated metabolic triad, in which the RPE maintains the canonical RPE65-dependent visual cycle, Müller glial cells support the cone-specific visual cycle through retinoid regeneration pathways, and RGR opsin provides an additional light-dependent photoisomerase activity in both RPE and specialized Müller cells, contributing to rapid chromophore recycling and cone visual pigment regeneration [12,13]. Disruptions in this cycle can lead to developmental abnormalities and progressive retinal degenerative disorders [14,15] as evidenced by inactivating mutations in key enzymes such as LRAT, RDH5, and RPE65, which impair chromophore synthesis and result in early-onset photoreceptor degeneration [16,17].

However, even a functional retinoid cycle can generate potentially harmful intermediates. Ocular retinoid homeostasis is tightly regulated, and its imbalance due to environmental factors or genetic predisposition can adversely affect retinal integrity [18,19]. Beyond the retina, vitamin A (retinol) and its primary metabolites, retinal and all-*trans*-retinoic acid (atRA), are collectively referred to as endogenous retinoids [20,21]. Retinal functions as an essential cofactor in the visual cycle, while atRA mediates the broader physiological roles of vitamin A, such as regulation of spermatogenesis, embryonic development, epithelial differentiation, and functions of the nervous and immune systems [22,23]. Vitamin A has limited solubility in aqueous media (~60 nM at physiological pH), which is lower than its concentrations in serum and most tissues [24]. Its poor aqueous solubility and often limited availability have driven the development of mechanisms to enhance its absorption, distribution, metabolism, and function.

To overcome these limitations, cells use specialized lipid-binding proteins that sequester and chaperone retinoids and other hydrophobic ligands. These proteins protect retinoids from nonspecific reactions within the cellular milieu while directing them toward specific metabolic enzymes and nuclear receptors [25,26,27]. Central to these processes are intracellular retinoid-binding proteins, including cellular retinol-binding proteins (CRBPs), cellular retinaldehyde-binding protein (CRALBP), and cellular retinoic acid-binding proteins (CRABPs). CRBPs and CRABPs belong to the intracellular lipid-binding protein (iLBP) family and share a conserved 10-stranded antiparallel β-barrel fold that encloses a hydrophobic ligand-binding cavity. In contrast, CRALBP is a structurally distinct retinoid-binding protein belonging to the CRAL-TRIO superfamily and adopts a characteristic Sec14-like fold. Despite these structural differences, these proteins function not merely as passive carriers but as active regulators that compartmentalize retinoids, direct their delivery to metabolic enzymes, and modulate receptor-mediated signaling [28]. In contrast, extracellular transport is mediated by serum retinol-binding protein 4 (RBP4), which maintains systemic retinoid distribution and homeostasis [29].

**Figure 1:**
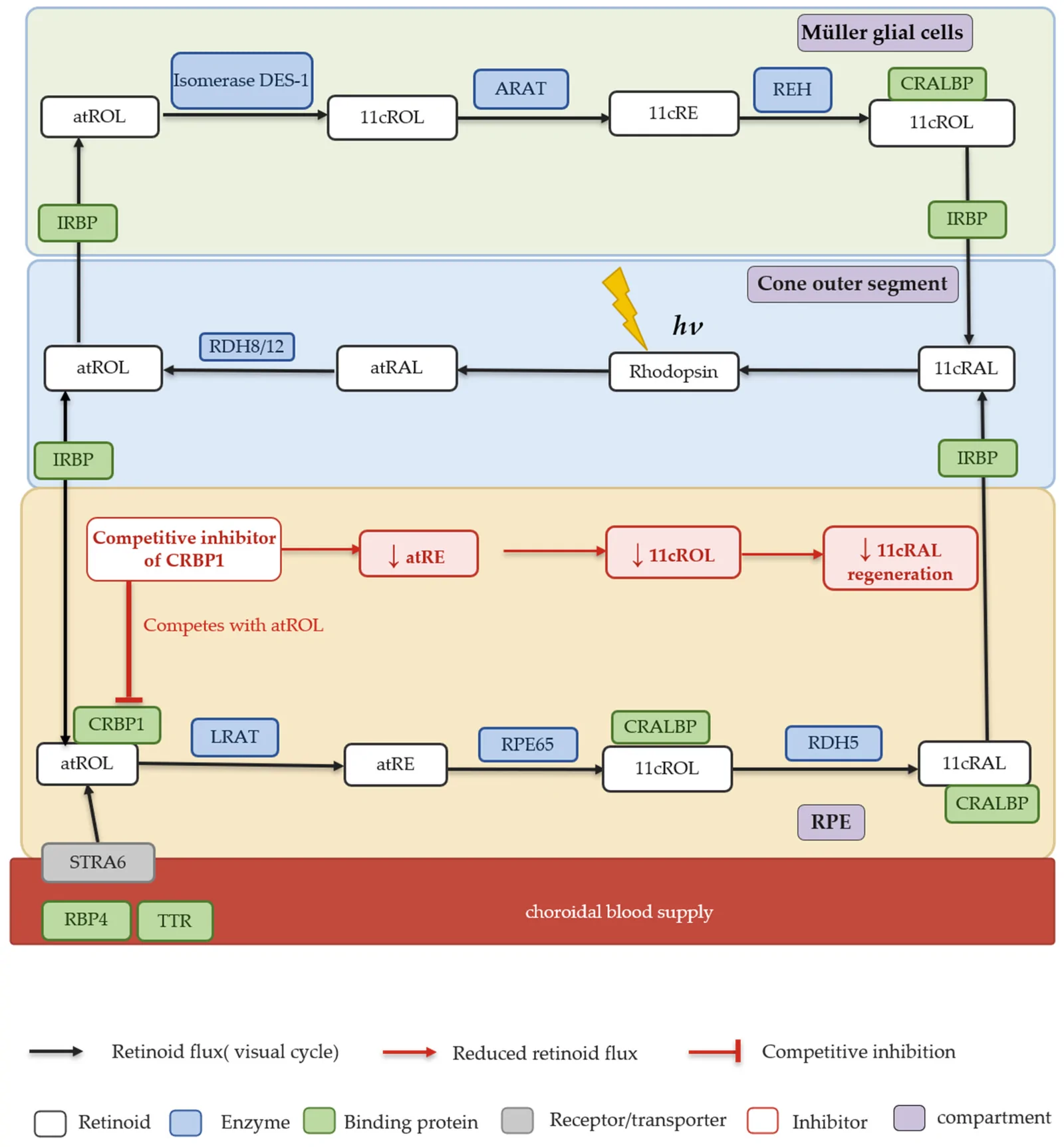
Schematic representation of the canonical RPE visual cycle and Müller cell-mediated intraretinal visual cycle, highlighting the roles of CRBP1 and CRALBP in retinoid trafficking. CRBP1 mediates intracellular trafficking of all-*trans*-retinol to LRAT for retinyl ester formation, whereas CRALBP binds and stabilizes 11-*cis*-retinol and 11-*cis*-retinal, promoting efficient chromophore regeneration and transport. Competitive inhibition of CRBP1 reduces retinol delivery to LRAT, thereby decreasing retinyl ester synthesis and limiting substrate availability for downstream visual cycle reactions. CRALBP dysfunction, in contrast, impairs stabilization and trafficking of 11-*cis*-retinoids, limiting chromophore regeneration and delaying rhodopsin reconstitution. Abbreviations: atROL, all-*trans*-retinol; atRAL, all-*trans*-retinal; atRE, all-*trans*-retinyl ester; RDH8/12, retinol dehydrogenase 8/12; 11cROL, 11-*cis*-retinol; 11cRAL, 11-*cis*-retinal; RDH5, retinol dehydrogenase 5; *hν*, photon; CRBP1, cellular retinol-binding protein 1; CRALBP, cellular retinaldehyde-binding protein; LRAT, lecithin:retinol acyltransferase; RPE65, retinal pigment epithelium-specific 65 kDa protein; RBP4, retinol-binding protein 4; TTR, transthyretin; IRBP, interphotoreceptor retinoid-binding protein; STRA6, stimulated by retinoic acid 6; RPE, retinal pigment epithelium; POS, photoreceptor outer segment.

Although RBP4 has been widely investigated as a therapeutic target [30], especially in the context of atRA-independent retinoid signaling in metabolic and ocular diseases, intracellular retinoid-binding proteins have received relatively less attention as tractable drug targets despite their close involvement in disease-relevant pathways. This functional and spatial distinction has significant pharmacological implications. Among retinoid-binding proteins, RBP4 has attracted the most pharmacological attention to date; clinical and preclinical inhibitors include the retinoid analogue fenretinide (4-hydroxyphenylretinamide) and the non-retinoid agents A1120 and BPN-14136 [30]. At the intracellular level, CRBP1 has also been targeted pharmacologically: abnormal cannabidiol (abn-CBD) was identified as a first-in-class competitive inhibitor with nanomolar affinity [31], and subsequent high-throughput screening identified non-retinoid scaffolds with improved drug-like properties [32]. Figure 1 illustrates the potential use of competitive inhibitors to reduce retinol flux through the visual cycle. This approach may represent a promising strategy for the development of therapeutics targeting intracellular retinoid-binding proteins. Nevertheless, systematic pharmacological targeting of intracellular retinoid-binding proteins remains at an early stage relative to their disease relevance.

These proteins are compelling candidates for structure-guided drug discovery due to hydrophobic binding cavities, ligand-selective conformational dynamics, and structurally defined interaction networks. Subtle modifications in binding pocket architecture and ligand specificity may be employed to develop selective small-molecule modulators that can change retinoid trafficking, block toxic intermediates, or modify retinoid-dependent signaling pathways. Advances in X-ray crystallography, computational docking, and molecular pharmacology now provide the structural and chemical tools to pursue this goal.

Existing reviews of retinoid biology have primarily focused on visual cycle enzymes, inherited retinal dystrophies, and retinoic acid-mediated transcriptional signaling. Although structural studies have characterized the ligand-binding properties of retinoid-binding proteins, their therapeutic potential remains insufficiently explored. This review addresses that gap by critically evaluating intracellular retinoid-binding proteins as emerging drug targets, integrating structural, biochemical, and pharmacological evidence to highlight how ligand specificity and binding pocket architecture can guide the development of selective small-molecule modulators.

## 2. Structural Biology of Intracellular Retinoid-Binding Proteins

### 2.1. CRBP Family (CRBP1 to 4)

The cellular retinol-binding protein (CRBP) family, comprising CRBP1 to CRBP4, belongs to the intracellular lipid-binding protein (iLBP) superfamily and mediates intracellular retinoid transport [33]. These small cytosolic proteins (~16 kDa) share a conserved fold but differ in ligand-binding properties, tissue distribution, and interactions with retinoid-metabolizing enzymes [34].

The iLBP fold consists of a flattened β-barrel formed by two nearly orthogonal five-stranded β-sheets, capped by a short α-helix-turn-helix “portal” region of two α-helices and connecting loops [35]. The portal governs ligand entry, and release distinguishes CRBPs from structurally related extracellular carriers such as Retinol-Binding Protein 4 (RBP4), which adopts an eight-stranded lipocalin fold [36,37]. Two portal α-helices and adjacent hairpin loops confer the conformational flexibility required for intracellular ligand trafficking [38,39].

Ligand binding in CRBPs follows an induced-fit model [40]. In the apo state, the portal of CRBP1 adopts an open conformation. Upon retinol binding, conformational rearrangements in helix II and adjacent loops promote closure of the binding-site portal, enclosing the ligand within the hydrophobic cavity. Reorientation of key residues, including Phe57, Tyr60, and Ile77, stabilizes the bound retinol and modulates ligand accessibility and release [40]. Retinol binds in an orientation inverted relative to serum RBP4. The polar hydroxyl group is buried deep within the barrel and forms hydrogen bonds with conserved polar residues, whereas the hydrophobic β-ionone ring remains near the portal [32,41]. This geometry underlies the high specificity of CRBPs for retinol (ROL) and retinal (RAL) [32].

CRBP isoforms differ in tissue distribution, ligand affinity, and physiological role [39]. CRBP1 is the most broadly expressed isoform and, within the retina, is most abundant in the retinal pigment epithelium (RPE) and Müller glia [42,43]. It binds all-trans-retinol with low-nanomolar (3 ± 2 nM) affinity [42,44,45]. Beyond chaperoning labile retinoids, CRBP1 accepts retinol delivered from plasma to the cytosol through the cell-surface receptor STRA6, which binds serum retinol-binding protein [46,47], facilitates retinol uptake from RBP4, and promotes its esterification [48]. Consistent with these roles, CRBP1-deficient mice show markedly reduced retinyl ester levels in hepatic stellate cells and the RPE. The lower ocular retinyl ester content is associated with an approximately 2-fold reduction in the rate of visual chromophore regeneration after light exposure, attributable to slower movement of vitamin A from photoreceptors to the RPE, where it is esterified [49,50]. In addition, apo-CRBP1 limits nonspecific oxidation by sequestering free retinol.

CRBP2 is enriched in intestinal enterocytes, where it supports dietary vitamin A absorption and esterification. Induction of jejunum Rbp2 expression in rats on a high-fat diet, together with elevated mucosal monoacylglycerol (MAG) levels and systemic metabolic disturbances in CRBP2-deficient mice, implicates the protein in lipid metabolism and neutral lipid signaling [51,52]. CRBP1 and CRBP2 share about 56% sequence identity [53]. Both bind all-trans-retinol, all-trans-retinal, and 13-cis-retinol, but neither binds 9-cis-retinol [42]. CRBP2 is the most selective isoform for all-trans-retinaldehyde, consistent with its role in the intestinal processing of retinal derived from β-carotene [42]. It retains high-affinity retinol binding (10–50 nM) but shows broader specificity, including binding of monoacylglycerols such as 2-arachidonoylglycerol. Thr51 and Val62 are the key residues that broaden ligand selectivity toward MAGs in CRBP2 [54] relative to the retinoid-specific CRBP1. These modifications within the binding pocket accommodate larger lipid species, indicating a role for CRBP2 in both retinoid and general lipid metabolism [54,55].

CRBP3 is expressed at lower levels and shows a more restricted distribution, primarily in the heart, skeletal muscle, adipose tissue, and lactating mammary gland [56,57]. It binds retinol with moderate affinity (Kd ≈ 60 nM) and appears to function under conditions of increased metabolic demand [58]. Evidence from knockout models indicates that CRBP3 contributes to retinol supply during lactation, as its absence leads to significantly reduced retinyl ester content in milk [59]. Data also indicate a role for CRBP3 in lipid metabolism, as its deficiency alters systemic lipid handling under metabolic stress [60,61].

Genetic studies suggest partial functional overlap between CRBP1 and CRBP3 in peripheral retinoid homeostasis. In Rbp3^-/-^ mice, CRBP1 protein levels were markedly elevated in adipose tissue and mammary gland [50,59]. In addition, in Rbp1^-/-^ mice, CRBP3 protein levels were elevated in tissues with endogenous CRBP3 expression, but not in tissues lacking CRBP3 expression [59]. These compensatory changes in protein expression suggest a potential ability of CRBP1 and CRBP3 to respond to the loss of the other protein; however, direct functional compensation has not been fully established. The distinct retinoid-related phenotypes observed in knockout models further indicate that CRBP1 and CRBP3 possess non-overlapping physiological roles. CRBP4 is the least characterized isoform, with low expression across multiple tissues, including the kidney, liver, and adipose tissue [62,63]. It exhibits weaker retinol-binding affinity (Kd ≈ 200 nM) [64] and lacks a clearly defined physiological function. Mouse CRBP4 is the only CRBP that binds 13-cis-retinol and 9-cis-retinol with Kd values comparable to that for all-trans-retinol [65].

Collectively, the CRBP family exemplifies how a conserved structural scaffold can support diverse biological functions through subtle variations in ligand-binding properties and tissue-specific expression. CRBP1 and CRBP2 exhibit the highest affinities for retinol and serve as primary regulators of intracellular retinoid trafficking, whereas CRBP3 and CRBP4 appear to play more specialized or context-dependent roles. High-resolution structures show that CRBP1 binds all-trans-retinol within a deeply buried hydrophobic cavity, where Lys40 and Gln108 stabilize its hydroxyl group, and conformational changes in portal residues control ligand access [40], whereas CRBP3 and CRBP4 carry a histidine residue that lowers ligand affinity [35,64].

Genetic evidence supports CRBP1 as a pharmacologically promising target. Rbp1^-/-^ mice show reduced retinyl ester storage in the RPE and a slight delay in visual cycle kinetics, but minimal serious retinal disease [40]. This suggests that partial inhibition of CRBP1 may alter retinoid flux, while remaining physiologically tolerable. Accordingly, CRBP1 has become the focus of efforts to identify competitive inhibitors, and it remains the only intracellular retinoid-binding protein actively pursued as a pharmaceutical target for retinal disease. However, despite detailed structural characterization, significant gaps remain in understanding the dynamic behavior of CRBPs, their interactions with non-retinoid ligands, and their broader roles in metabolic regulation.

### 2.2. CRALBP

Cellular retinaldehyde-binding protein (CRALBP), encoded by the RLBP1 gene, is a ~36 kDa [66] soluble cytosolic protein belonging to the CRAL-TRIO domain family, which includes lipid transfer proteins such as Sec14 and α-tocopherol transfer protein (α-TTP) [66,67]. CRALBP was originally isolated from bovine retina in complex with endogenous 11-cis-retinal and 11-cis-retinol, highlighting its role as a high-affinity carrier of visual cycle retinoids [38].

CRALBP adopts a characteristic Sec14-like fold with a deep hydrophobic ligand-binding cavity capped by a dynamic α-helical lid that regulates ligand accessibility [68,69]. Compared with the relatively compact β-barrel cavity of CRBP1, the CRALBP pocket is larger and conformationally flexible, allowing it to sequester and stabilize bulky 11-cis-retinoids in the aqueous cytosolic environment while maintaining controlled ligand exchange.

Photoaffinity labeling of recombinant human CRALBP identified eight residues (Tyr179, Phe197, Cys198, Met208, Lys221, Met222, Val223, and Met225) within the ligand-binding cavity, expanding the number of known interaction residues to twelve, and hydrogen/deuterium exchange analysis confirmed the predominantly hydrophobic character of this pocket [70]. The cavity is enriched in residues such as Trp165, Tyr179, Phe197, Met222, and Met225, which stabilize the retinoid chromophore through close-range hydrophobic interactions (4 to 5 Å) [68,71]. Additional residues including Cys198, Gln210, Lys221, and Val223 provide secondary polar and structural contacts that further stabilize ligand binding [72]. Modeling places Met208 on a flexible loop, whereas distal residues such as Arg233 and Trp244, particularly within the conserved helical region, govern the opening and closing of the cavity, and thereby control ligand accessibility and exchange dynamics [68,69]. Crystallographic analyses suggest that this lid motion modulates ligand exchange kinetics and that the lid region may serve as an allosteric site for pharmacological intervention.

CRALBP is highly expressed in both the RPE and Müller glia [73], where it facilitates the vectorial transfer of 11-cis-retinoids between visual cycle enzymes during chromophore regeneration [74,75,76]. By stabilizing 11-cis-retinoids and preventing their spontaneous isomerization or degradation, it ensures a continuous supply of chromophore required for both rod- and cone-mediated phototransduction [77]. Although CRALBP does not directly participate in nuclear receptor-mediated retinoid signaling, it is essential for maintaining retinal retinoid homeostasis [78]. The disease-associated R234W mutation induces cascading conformational changes that increase pocket packing density, resulting in tighter ligand binding, slower ligand release, and the impaired retinal metabolism that underlies Bothnia dystrophy [79,80]. These findings indicate that relatively subtle structural perturbations within the CRALBP pocket can markedly alter retinoid trafficking dynamics, supporting the feasibility of pharmacological modulation of CRALBP function.

In preclinical studies, subretinal delivery of an AAV8-hRLBP1 vector to Rlbp1^-/-^ mice improved the rate of both cone and rod dark adaptation [81]. CRALBP deficiency does not abolish visual cycle function but alters its kinetics. In Rlbp1^-/-^ mice, 11-cis-retinal levels are markedly lower than in wild-type (WT) mice, and recovery of visual sensitivity following visual pigment bleaching is appreciably slowed [82]. Structural and mutational studies indicate that Gln210 and Lys221 mediate retinoid binding by CRALBP likely through noncovalent contacts that stabilize the ligand within the binding cavity [70].

### 2.3. CRABP1 and CRABP2

CRABPs are small (16 kDa), soluble proteins of the intracellular lipid-binding proteins (iLBPs) family, defined by a β-barrel formed from two orthogonal five-stranded β-sheets that encloses a large, deep binding cavity. Two short α-helices form a cap-like portal that regulates ligand entry and exit [83]. Ligand entry is proposed to occur through a dynamic portal formed by the βC–D loop, the βE–F loop, and the N-terminal region of helix II [84]. Similar portal-mediated exchange mechanisms have been characterized across the iLBP family by X-ray crystallography, mutational analysis, and multidimensional NMR, underscoring the role of conformational flexibility in regulating ligand accessibility [85,86]. NMR and mass spectrometry show that the portal region of apo-CRABP1 has markedly greater backbone flexibility than the rest of the protein, whereas retinoic acid binding stabilizes this region [84,87].

Both CRABP1 and CRABP2 bind all-trans-retinoic acid (atRA) with low-nanomolar affinity, functioning as high-affinity intracellular carriers of retinoic acid. At the deepest part of the cavity, a conserved polar network anchors the carboxylate moiety of atRA through hydrogen bonding and electrostatic interactions. This network comprises Arg112, Arg132, and Tyr134 in CRABP1 and Arg112, Arg133, and Tyr135 in CRABP2 [88,89]. These residues are critical determinants of ligand specificity and high-affinity binding.

Although CRABP1 and CRABP2 share highly conserved ligand-binding cavities, which makes isoform-selective drug design challenging, subtle differences in the dynamics of the helical cap and portal may offer opportunities for selective allosteric modulation. In particular, the portal of CRABP2 adopts conformations that facilitate exposure of its nuclear localization signal and interaction with retinoic acid receptors, features largely absent in CRABP1. The two isoforms also diverge functionally in retinoic acid trafficking and signaling. CRABP2 mediates nuclear delivery of retinoic acid to RAR/RXR complexes, and thereby shapes transcriptional programs, whereas CRABP1 channels retinoic acid toward catabolic pathways and participates in non-genomic signaling through signalosome-mediated modulation of CaMKII and the RAF-MEK-ERK pathway [33].

Through these non-genomic mechanisms, CRABP1 acts as a cytosolic regulator that confers neuroprotection by modulating kinase signaling pathways, including CaMKII and RAF-MEK-ERK [90]. In excitable cells, it negatively regulates CaMKII by limiting calmodulin-dependent activation, preventing aberrant Ca^2+^-driven kinase overactivation and preserving signaling homeostasis. In parallel, it modulates RAF-MEK-ERK signaling in a cell-type-dependent manner, promoting pro-survival ERK activation while suppressing dysfunctional Ras-mediated overactivation. Consistent with these roles, CRABP1-deficient models exhibit motor neuron degeneration and cardiomyopathy [91]. Small-molecule ligands such as C32 and C4 selectively modulate CRABP1-dependent CaMKII signaling and protect against excitotoxic neuronal injury [91]. Together, these findings establish CRABP1 as a regulator of signaling homeostasis and highlight its potential as a therapeutic target for kinase-driven diseases that do not depend on nuclear retinoid signaling [92,93]. Table 1 provides a comparative summary with distinct ligand specificities, tissue distributions, and biological functions of the CRBP, CRALBP, and CRABP families.

## 3. Disease Relevance

### 3.1. Inherited Retinal Dystrophies

Biallelic mutations in the *RLBP1* gene disrupt the canonical visual cycle and cause a clinically heterogeneous spectrum of autosomal recessive inherited retinal dystrophies, including Bothnia dystrophy, retinitis pigmentosa, retinitis punctata albescens, fundus albipunctatus, and Newfoundland rod-cone dystrophy [82,102,103]. The encoded protein, CRALBP, is a high-affinity carrier of 11-*cis*-retinol and 11-*cis*-retinal in the RPE and Müller glia, where it facilitates retinoid trafficking during visual chromophore regeneration and limits the spontaneous isomerization and aldehyde toxicity of reactive retinoids. Loss of CRALBP markedly impairs chromophore regeneration, resulting in delayed dark adaptation, early-onset nyctalopia, and accumulation of retinoid-derived subretinal deposits.

Transcriptional studies show that retinal-specific regulatory elements, such as the PCE-1 region, tightly control CRALBP expression, underscoring its importance for retinal homeostasis [104]. Whole-exome sequencing of affected individuals with compound heterozygous variants identified c.25C>T (p.Arg9Cys) and c.286_297del (p.Phe96_Phe99del) in *RLBP1*. Segregation analysis confirmed that the father was heterozygous for the c.25C>T (p.Arg9Cys), whereas the mother carried the c.286_297del (p.Phe96_Phe99del) variant. Quantitative autofluorescence (qAF), a non-invasive imaging biomarker of RPE lipofuscin accumulation and visual-cycle-associated metabolic stress, was lower in the mother than in the father. However, qAF primarily reflects RPE bisretinoid content and alterations in visual-cycle metabolism rather than directly assessing photoreceptor function or visual performance. Therefore, differences in qAF should be interpreted cautiously, as qAF measurements can be influenced by multiple factors, including age, lens transmittance, axial length, and ethnicity. Comprehensive assessment of retinal function using complementary approaches, such as electroretinography, visual acuity testing, dark adaptation, or microperimetry, is required to determine the functional consequences of specific genetic variants [78].

Because Arg9 lies in the disordered N-terminal region of CRALBP, which is unresolved in crystal structures, a direct structural mechanism cannot be determined [78]. Nonetheless, replacing the positively charged arginine with cysteine may alter local electrostatic interactions, protein folding, and pH-dependent activity.

By contrast, the Phe96–Phe99 deletion removes one positively charged residue (Arg98) and three hydrophobic residues (Phe96, Leu97, and Phe99) from the α-helical domain of CRALBP [78]. Although this region lies approximately 25 Å from the retinal-binding pocket [79], structural analyses suggest that the deletion may induce long-range conformational rearrangements that compromise the integrity of the binding site. Deletion of residues 96 to 99 is proposed to reposition Arg234 by abolishing its contact with Glu51, a 2.7 Å interaction observed in the native crystal structure, thereby perturbing the retinal-binding pocket. Loss of Phe99-mediated contacts near Pro232 may likewise alter the conformation of Arg234, a residue critical for normal CRALBP function [78]. The disease-associated Arg234Trp mutation destabilizes the ligand-binding pocket, impairs 11-*cis*-retinal handling, and reduces functional activity approximately 5-fold, directly linking CRALBP conformational stability to visual-cycle dysfunction [79,105].

### 3.2. Oncological and Metabolic Roles of CRABP Isoforms

Cellular retinoic acid-binding proteins (CRABP1 and CRABP2) have emerged as key regulators of retinoic acid (RA) signaling, with unique and often opposing functions in cancer biology and metabolic regulation [106]. CRABP2 primarily delivers RA to nuclear retinoic acid receptors (RARs) and thereby regulates transcriptional programs, whereas CRABP1 supports non-genomic signaling through cytoplasmic protein–protein interactions and signalosome formation. Beyond its role in retinoid transport, CRABP2 functions as a critical mediator of retinoic acid signaling by directly delivering RA to nuclear retinoic acid receptors (RARs). This ligand channeling enhances RAR-dependent transcription of genes involved in cell-cycle regulation and apoptosis, including the cell-cycle regulator BTG2 and pro-apoptotic targets, thereby contributing to suppression of mammary carcinoma cell proliferation [107,108,109]. This growth-suppressive function of CRABP2 is counterbalanced by FABP5, which preferentially channels RA to PPARβ/δ signaling and promotes cell survival. Thus, the relative abundance and activity of the CRABP2/RAR and FABP5/PPARβ/δ pathways can determine whether RA elicits anti-proliferative or pro-survival responses [110]. Accordingly, ectopic CRABP2 expression suppresses mammary carcinoma growth in vivo, while reduced CRABP2 expression, including promoter hypermethylation-mediated silencing, has been linked to impaired retinoic acid responsiveness [111]. Notably, CRABP2 function is context-dependent, with both tumor-suppressive and pro-tumorigenic roles reported across cancers, including pro-metastatic effects in non-small-cell lung and ovarian carcinomas, highlighting the importance of tissue-specific signaling networks.

Mutagenesis studies identify Lys102 as the sole RA–dependent SUMOylation site in CRABP2, and its substitution abolishes nuclear translocation, impairing CRABP2-mediated activation of RARs. CRABP2 increases tumor cell migration and invasion by activating pathways such as PI3K/AKT and MAPK and by inducing extracellular matrix components such as LAMB3 [112]. In vivo studies further show that CRABP2 promotes tumor growth and metastasis through the ROS-Src signaling axis, linking oxidative stress to metastatic progression [113]. Beyond the tumor cells themselves, CRABP2 shapes the tumor microenvironment by regulating cancer-associated fibroblast activity and immune checkpoint pathways, and higher expression correlates with poor prognosis and reduced response to immunotherapy [114].

CRABP2 also sequesters docetaxel a taxane-based anticancer agent, with an affinity exceeding that of its intended target, providing a potential mechanism of chemotherapeutic resistance, with an affinity exceeding that of the drug’s intended target, providing a mechanism for chemotherapeutic resistance [115,116]. It suppresses apoptosis and promotes cancer cell survival under chemotherapeutic stress. In MDA-MB-231 and BT549 cells, CRABP2 knockdown reduces proliferation and enhances docetaxel-induced apoptosis [115]. This evidence reframes resistance not only as a signaling phenomenon but also as a buffering effect, in which intracellular proteins influence drug efficacy through competitive binding.

CRABP1 regulates cell behavior through non-genomic signaling. The CRABP1-RA activates ERK1/2 and inhibits the G1-S cell cycle transition, and it also influences exosome secretion and intercellular communication within the tumor microenvironment [117]. The balance between apo- and holo-CRABPs acts as a sensor of intracellular retinoid status, regulating RA flux and catabolism through cytochrome P450 enzymes such as CYP26A1, which convert RA to inactive polar metabolites [98].

CRBP1, by contrast, governs retinoid metabolism by directing dietary retinol toward retinyl ester formation, maintaining cellular homeostasis and minimizing oxidative damage. Tumorigenic epithelial cell lines lacking CRBP1 generate atRA from retinol at only about half the rate of non-tumorigenic cells, and *Rbp1*-null mice have roughly 40% less atRA in mammary tissue than WT animals [117].

### 3.3. CRBP1 in Retinal Degeneration and Hepatic Retinoid Storage

Within the retina, CRBP1 is highly expressed in the retinal pigment epithelium (RPE), where it regulates the intracellular trafficking of all-*trans*-retinol, the substrate required for visual chromophore regeneration. Recent studies indicate that CRBP1 is the primary intracellular transporter of retinol in ocular tissues, placing it upstream of the visual cycle and phototransduction [32]. More recent work suggests that CRBP1 does more than deliver retinol. It also modulates the rate and selectivity of retinoid flux into competing metabolic pathways. Structural studies show that CRBP1 undergoes ligand-induced conformational changes that stabilize retinol within a protected binding pocket, preventing nonspecific oxidation and selectively delivering retinol to enzymatic partners. This controlled release mechanism determines whether retinol is directed toward chromophore regeneration, storage, or degradation [40]. Inhibition or inactivation of CRBP1 reduces bisretinoid accumulation and protects against light-induced retinal degeneration, indicating that excessive or uncontrolled retinoid flux contributes to retinal toxicity [118].

After dietary intake, retinoids reach the liver as chylomicron remnants, which are taken up by hepatocytes, the primary entry point for postprandial vitamin A [119,120]. Within hepatocytes, retinyl esters are hydrolyzed to retinol, which is then released into the circulation bound to RBP4 or transported to hepatic stellate cells (HSCs), the principal storage site of vitamin A [121]. Although HSCs comprise only a small proportion of liver cells, they store about 70–90% of hepatic retinoids as retinyl esters in specialized lipid droplets [122,123], playing a disproportionate role in systemic vitamin A homeostasis. The exact mechanism by which retinol is transferred from hepatocytes to stellate cells remains unclear [124]. Experiments in RBP4-deficient mice show that hepatic retinyl ester storage is largely unaffected, indicating that this intercellular transport does not depend on RBP4 [125].

CRBP1 deficiency reduces retinyl ester (RE) accumulation in HSCs by about 50%. This reduction reflects both decreased synthesis and a roughly 6-fold faster turnover, without corresponding changes in the levels of RE metabolizing enzymes, and it most likely results from impaired delivery of ROL to lecithin: retinol acyltransferase (LRAT) [50,125]. CRBP1 is therefore indispensable for efficient retinol handling and RE accumulation in stellate cells.

LRAT is solely responsible for vitamin A esterification in HSCs and for the formation of their lipid droplets [16,126]. In homozygous *Lrat* knock-out (*Lrat*^-/-^) mice, RE is undetectable in liver extracts, ruling out contributions from DGAT1 or other acyl-CoA-dependent transferases to hepatic retinol esterification. The amount of RE stored in HSCs correlates strongly with dietary vitamin A and carotenoid supply, underscoring the regulatory role of these cells in retinoid homeostasis [126,127].

## 4. Druggability Assessment

### 4.1. Binding Pocket Analysis and Selectivity Landscape Within the iLBP Family

CRBPs and CRABPs belong to the intracellular lipid-binding protein (iLBP) family and share a conserved 10-stranded antiparallel β-barrel fold that encloses an internal hydrophobic ligand-binding cavity [38,128]. In contrast, CRALBP belongs to the structurally distinct CRAL-TRIO superfamily and adopts a Sec14-like fold with a larger, conformationally flexible ligand-binding pocket. Despite these structural differences, the pharmacological tractability of these retinoid-binding proteins is strongly influenced by the size, shape, polarity, and accessibility of their ligand-binding pockets, as well as by the presence of gating residues that regulate ligand binding and release [128,129].

Human CRBP1 adopts the conserved iLBP fold, a single-domain structure in which 10 antiparallel β-strands assemble into a flattened β-barrel that encloses the retinoid-binding cavity [35]. Access to this cavity is asymmetric. One end is closed by β-sheet residues and the N-terminus, whereas the opposite end forms a dynamic portal made up of two short α-helices and the βC–βD and βE–βF hairpin turns, which together govern ligand entry and release [40]. The cavity is deeply buried and amphipathic, combining a hydrophobic region formed by residues such as Phe57, Leu74, Tyr60, and Ile77, which tightly encapsulate the retinoid, with a hydrophilic region that includes Gln108 and Lys40 and stabilizes the ligand through hydrogen bonds. Together, these features enable CRBP1 to selectively bind, transport, and release retinoids within the cell [32,40,41].

The presence of structured water networks further enhances ligand-binding flexibility and may support the development of diverse chemical scaffolds. Ligand-induced conformational rearrangements, together with internal hydrogen-bonding networks, help stabilize the bound state, indicating a structurally defined and energetically favorable binding environment. High-resolution co-crystal structures of non-retinoid inhibitors show that they occupy the same cavity as retinol while forming alternative hydrogen bonds, including contacts mediated by ordered water molecules [40].

The discovery of ligands with nanomolar affinity, such as abnormal-cannabidiol (abn-CBD), shows that the pocket can support high-affinity binding beyond its native substrate, an important requirement for a promising therapeutic target [40]. It was identified as a competitive inhibitor of CRBP1 in a fluorescence-based high-throughput screen (HTS). Although abn-CBD shows little structural resemblance to all-*trans*-retinol, the two ligands bind CRBP1 with similar affinity. Its scaffold comprises a cyclic monoterpene unit linked to a dihydroxybenzene (resorcinol) ring that carries a pentyl side chain.

Crystallography revealed the key ligand–protein interactions underlying the favorable binding of abn-CBD to CRBP1. The cyclohexenyl ring is held within the hydrophobic pocket by Phe16, Tyr19, Leu20, Phe57, Arg58, Tyr60, Ile77, and Met119. Ala33 hydrogen bonds with the ortho hydroxyl (relative to the cyclohexenyl ring), whereas the para hydroxyl forms hydrogen bonds with Gln128 and a water molecule. The pentyl moiety shares the same site as the all-*trans*-retinol polyene chain and appears to have a minimal role in binding affinity. The replacement of the pentyl chain with a methyl moiety, as mentioned in abnormal-cannabidiorcin (abn-CBDO), lowered inhibitory efficacy by 4-fold. Selectivity within the iLBP family is the central pharmacological challenge. Comparative analysis of CRBP isoforms shows that minor residue differences can critically determine ligand selectivity. In CRBP2, a single substitution (Pro38→Gln) introduces steric hindrance that blocks abn-CBD binding, whereas smaller substitutions of Phe16/Met and Ile77/Leu in CRBP3 and Leu20/Met, Phe57/Leu, and Ile77/Leu in CRBP4 preserve pocket architecture and allow binding similar to CRBP1 [31]. These data indicate that small structural differences can regulate ligand accessibility without altering the overall fold, supporting the feasibility of isoform-selective drug design within the CRBP family. The combination of tight sequestration and a defined pocket architecture makes CRBP1 a suitable candidate for small-molecule targeting, although its limited, largely non-polar cavity may constrain ligand diversity and design.

In CRALBP, the CRAL-TRIO ligand-binding spans approximately residues Arg120 to Lys294. CRALBP has a two-domain structure, comprising an N-terminal all-helical domain and a C-terminal domain that forms the ligand-binding cavity. The N-terminal region (residues 66 to 119) consists of four antiparallel α-helices, three of which form a tripod-like motif while the final helix links and stabilizes the two domains. The C-terminal domain contains a structured binding pocket built around a central β-sheet, enclosed by α-helices, and capped by a helical loop that regulates ligand access. This arrangement and the partially enclosed cavity suggest that ligand binding in CRALBP is structurally coordinated and gated, offering the opportunity to target ligand entry and release rather than competitive binding alone.

Molecular dynamics simulations showed that acidic phospholipids induce allosteric conformational changes in CRALBP that facilitate 11-*cis*-retinaldehyde release, identifying conformational dynamics and membrane-interacting regions as potential therapeutic targets [130]. These findings suggest that allosteric regulation of the membrane-interacting surface of CRALBP may offer an alternative to direct orthosteric targeting of the tightly sealed retinoid-binding cavity. In retinal degenerative diseases, therefore, the exposed cationic phospholipid-binding surface may represent a previously unexplored druggable target for modulating retinoid release and visual cycle activity [130].

To improve chromophore stability and controlled retinoid delivery, Van de Werken et al. engineered a disulfide-locked CRALBP mutant (A212C/T250C), in which Ala212 and Thr250 were replaced with cysteine to form a reversible disulfide bridge across the mobile ligand-binding gate. This bridge protects 9-*cis*-retinal during extracellular transport and enables tunable chromophore release under reducing conditions [131]. The engineered carrier improved photostability, restored scotopic electroretinogram (ERG) responses in *Rpe65*^-/-^ retinas, and accelerated dark adaptation in vivo, demonstrating the therapeutic potential of CRALBP-based chromophore delivery systems [131]. The R234W Bothnia dystrophy mutation induces a domino-like conformational rearrangement that propagates approximately 15 Å from the protein surface to the ligand-binding cavity. This rearrangement increases packing within the binding pocket, stabilizes the 11-cis-retinal complex, and impairs ligand release [79].

In CRBP1, ligand binding is governed by a dynamic portal mechanism rather than by static cavity saturation. In the apo state, the portal region is flexible and partially occludes the cavity, with key residues projecting into the binding site and limiting access. Upon ligand engagement, conformational rearrangements in this region open the cavity to admit the ligand, after which a closed conformation is stabilized that encloses the ligand [40]. This induced-fit mechanism has important pharmacological implications. Binding affinity depends strongly on a ligand’s ability to stabilize the closed portal conformation, rather than merely on internal hydrogen bonding [40].

The crystal structures of CRABP1 and CRABP2 reveal a more open ligand access portal, with the β-ionone ring readily accessible to enzymes that modify the C4 and C18 positions. At the deepest part of the binding cavity, conserved residues (Arg112, Arg132, and Tyr134 in CRABP1 and Arg112, Arg133, and Tyr135 in CRABP2) stabilize the carboxylate of all-trans retinoic acid through hydrogen bonds and electrostatic interactions, together with a conserved water molecule [88].

Studies of the CRABP isoforms show that CRABP2 specifically channels retinoic acid to RAR through direct protein–protein interactions. Structural and mutational analyses identified a distinct surface region comprising Gln75, Pro81, and Lys102 that is essential for CRABP2 binding to RAR and for activation of retinoid-dependent transcription [132]. Transferring these residues into CRABP1 conferred CRABP2-like signaling activity, indicating that small structural differences between the isoforms could enable isoform-specific therapeutic modulation. High-resolution co-crystal structures further show that synthetic retinoids such as DC645 and DC479 bind within the canonical retinoic acid–binding cavity in a manner closely resembling all-*trans*-retinoic acid. DC645 produced a disease-modifying effect in neurodegenerative models, reinforcing the pharmacological and structural druggability of the CRABP isoforms [133].

### 4.2. Known Small-Molecule Modulators

#### 4.2.1. The First-in-Class Inhibitor: Abnormal Cannabidiol (abn-CBD)

The identification of abn-CBD as a CRBP1 inhibitor by Silvaroli et al. [31] established proof of concept for pharmacologically targeting this protein. abn-CBD is a synthetic, non-psychoactive derivative of plant cannabidiol that lacks activity at the cannabinoid receptors CB1 and CB2 [134]. Its interaction with CRBP1 was discovered by high-throughput screening (HTS) of a ~1000-compound bioactive lipid library using a fluorescence resonance energy transfer (FRET)-based fluorescence displacement assay. FRET assays rely on energy transfer between a donor and an acceptor in close proximity, producing a measurable change in emitted energy [135]. Competitive binding of an inhibitor displaces the retinoid from the binding pocket, increasing the distance between the intrinsic tryptophan donor and the retinoid acceptor and reducing the FRET signal.

**Figure 2:**
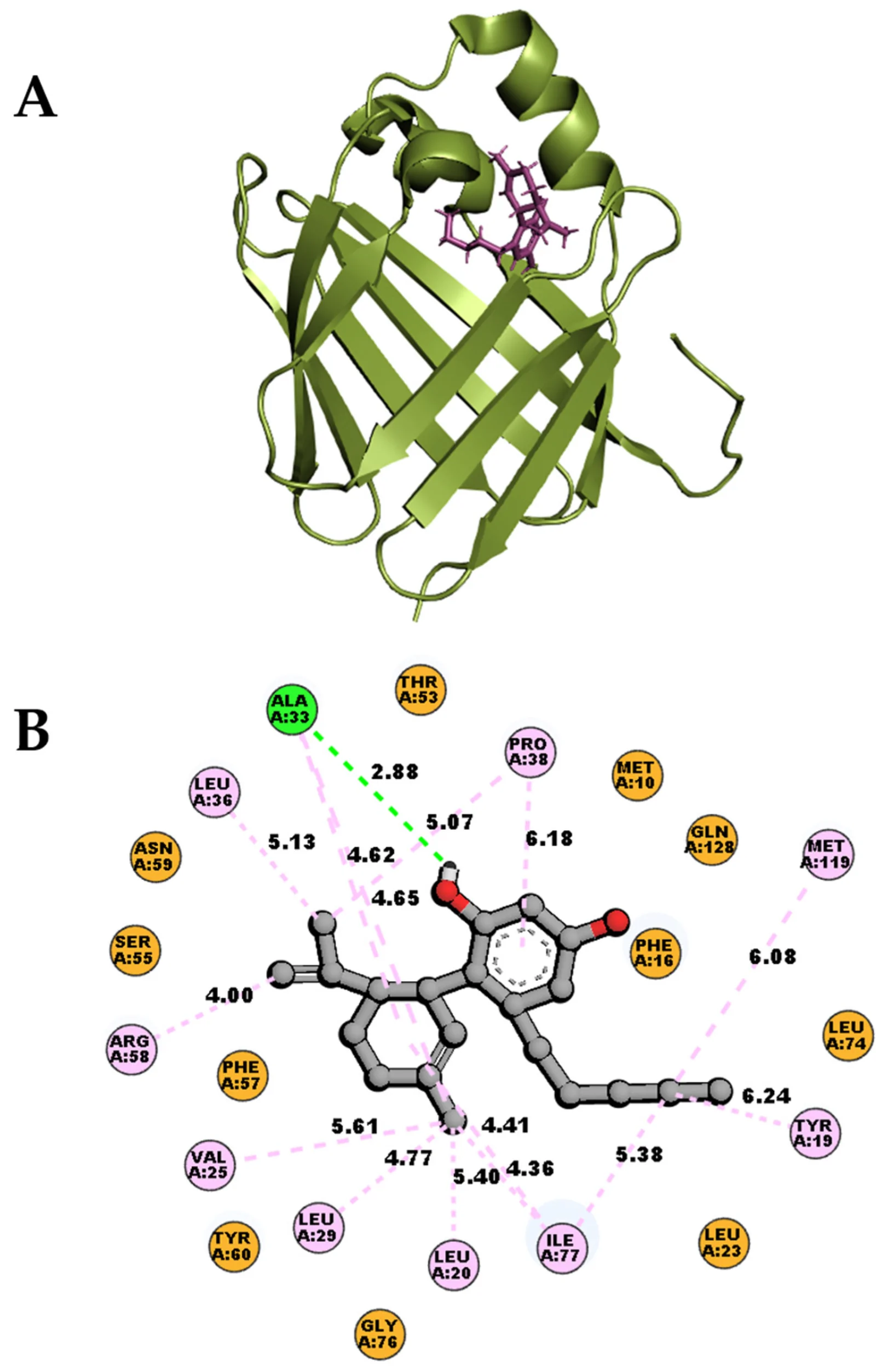
(**A**) Crystal structure of CRBP1 in complex with abn-CBD (PDB ID 6E5L, 1.17 Å). abn-CBD (magenta sticks) binds within the β-barrel near the α-helical portal. (**B**) Close-up of the binding pocket showing abn-CBD (gray ball-and-stick) and the surrounding residues, with hydrogen bonds as green dashed lines and hydrophobic contacts as pink dashed lines. Distances are shown in Å. Panel (**A**) was prepared in PyMOL Molecular Graphics System version (3.1.6.1); panel (**B**) was generated with BIOVIA Discovery Studio Visualizer 2025 version (v25.1.0.24284).

The crystal structure of CRBP1 in complex with abn-CBD at 1.17 Å resolution (PDB 6E5L) revealed a binding mode that overlaps with atROL but occupies regions of the cavity not contacted by the natural ligand (Figure 2A). The cyclohexenyl ring sits in the same hydrophobic portal cleft as the β-ionone ring of retinol, engaging Phe16, Tyr19, Leu20, Phe57, Arg58, Tyr60, Ile77, and Met119 through van der Waals contacts, and the aliphatic pentyl chain follows the polyene-binding trajectory (Figure 2B). The distinguishing feature is the benzenediol (resorcinol) moiety, which extends into a sub-pocket. Its ortho-hydroxyl forms a single hydrogen bond with the carbonyl oxygen of Ala33, a helix II residue, while its para-hydroxyl participates in a hydrogen-bond network with the side chains of Asn13, Lys40, and Gln128, with at least four ordered water molecules [40].

By contacting Ala33, abn-CBD anchors itself to the conformationally dynamic α-helix II and stabilizes the portal in its closed conformation. The B-factor of the portal main chain decreases from 30.6 Å^2^ in apo-CRBP1 to 9.8 Å^2^ in the abn-CBD complex, comparable to 10.5 Å^2^ in the atROL complex, indicating that abn-CBD stabilizes the closed portal as effectively as the natural substrate despite its distinct scaffold [31]. abn-CBD achieves nanomolar affinity (*Ki* ~67 nM) [40] and is roughly 150-fold more potent than the next-best scaffold identified by the same screen.

#### 4.2.2. Structure–Activity Relationships Derived from abn-CBD Analogues

A systematic structure–activity relationship (SAR) analysis of three abn-CBD analogues, together with limonene as a control, defined the individual contributions of its key pharmacophoric features (the cyclohexenyl ring, the pentyl side chain, and the benzenediol moiety) to binding affinity and functional activity. In abn-CBDO, in which the pentyl chain is shortened to a methyl group, *Ki* increases 4.3-fold relative to abn-CBD, indicating that the aliphatic chain makes substantial van der Waals contacts in the hydrophobic cavity and cannot be shortened without loss of affinity. In CBDO, in which the para- and ortho-hydroxyl positions are reversed, *Ki* increases ~6.3-fold (~1.7 μM) [31], reflecting reduced binding affinity. In the co-crystal structure (PDB 6E6M), the para-hydroxyl shifts to align with Gln128 and the ordered water network, whereas the ortho-hydroxyl can no longer interact with Ala33. This asymmetric loss of affinity underscores the central role of the para-hydroxyl-mediated hydrogen-bond network as the primary anchoring interaction for abn-CBD. In O-1918, in which both hydroxyls are methylated and therefore cannot hydrogen-bond, no binding is detectable up to 0 to 10 μM [31], indicating that hydrogen bonding by the aromatic ring is essential for meaningful affinity. Limonene shows no binding, indicating that the aromatic and aliphatic features are jointly required for CRBP1 interaction.

These findings define a three-component pharmacophore for high-affinity CRBP1 inhibition. First, a bulky hydrophobic moiety occupies the portal cleft through van der Waals interactions, analogous to the β-ionone ring of atROL. Second, an aliphatic chain aligns with the polyene-binding trajectory. Third, a polar group forms hydrogen bonds with portal-region residues, particularly within α-helix II. Loss of the hydrogen-bonding element causes a marked decrease in affinity despite favorable hydrophobic interactions.

#### 4.2.3. Novel Non-Retinoid Scaffolds Identified by HTS

The study by Plau et al. was motivated by the suboptimal pharmacokinetic profile of abn-CBD, in particular its high lipophilicity (logP~6.5) and poor oral bioavailability [32]. To address this, the authors performed an expanded high-throughput screening (HTS) of a drug-like library of 45,840 compounds using a FRET-based displacement assay with abn-CBD as the reference ligand [32]. After stepwise filtering, including exclusion of spectroscopic interference, fluorescence titration filtering that excluded spectroscopic interference and applied fluorescence titration validation, and co-crystallization as a structural filter, four compounds were confirmed as CRBP1 inhibitors.

Three of the four initially identified hit compounds (inhibitors 1 to 3) are structurally related, each containing a 1,2,4-oxadiazole ring linked through a tertiary amine to either a thiophene (inhibitor 1) or a pyrazole ring (inhibitors 2 and 3), with a bulky hydrophobic group at the 3-position of the oxadiazole, either a 4-methylphenyl cyclopentyl (inhibitors 1 and 2) or a diphenyl methyl (inhibitor 3). Inhibitor 4 has a distinct structure, with a sulfonyl group attached to a methoxy-tetrahydronaphthalene moiety and a 4-(hydroxymethyl)piperidine. Compounds 1–4 were identified directly through high-throughput screening (HTS). Subsequent structure–function analysis, integrating biochemical characterization, structural comparison, and evaluation of ligand–CRBP1 interactions, enabled the selection and evaluation of additional related derivative compounds 5 and 6. These compounds were further characterized together with compounds 1–4 to define the structural determinants governing CRBP1 binding and inhibition [32].

Subsequent structural and biochemical analyses were performed on six inhibitors (compounds 1–6). All six inhibitors showed *Ki* of 7.1 to 10.7 μM, roughly 100 to 160-fold weaker than abn-CBD. Their lower lipophilicity (logP 1.4 to 5.0), however, indicates improved drug-like properties relative to abn-CBD. High-resolution X-ray crystal structures (1.13 to 1.85 Å) of the complexes show that, although these ligands occupy the same cavity as atROL and abn-CBD, they engage the portal region through distinct interaction modes [32].

## 5. Therapeutic Strategies

### 5.1. Gene Therapy

The eye is an attractive target for gene therapy because of its immune-privileged environment maintained by the blood–retinal barrier, which limits inflammatory responses. Its small, enclosed structure allows effective treatment at low doses with minimal systemic exposure, and the paired-eye anatomy enables direct comparison with the untreated eye. The eye is also readily accessible for precise delivery (for example, subretinal or intravitreal injection) and for non-invasive monitoring of therapeutic outcomes [136].

Retinal gene therapy has rapidly become a leading strategy for inherited retinal diseases (IRDs), driven by advances in genetic characterization and by the suitability of the eye as a therapeutic target. Nearly 300 causative genes have been identified [137]; most IRDs are monogenic disorders affecting the RPE and photoreceptors, making them strong candidates for gene replacement or augmentation. Clinical progress has been especially clear with adeno-associated virus (AAV)-mediated delivery, exemplified by voretigene neparvovec (Luxturna), which restores functional RPE65 expression and improves visual outcomes in Leber congenital amaurosis. Numerous trials are also exploring AAV-based therapies for conditions such as choroideremia and retinitis pigmentosa, alongside emerging genome-editing approaches such as CRISPR/Cas9 [138].

AAVs are small, non-enveloped (~25 nm) single-stranded DNA viruses (~4.7 kb) that are widely used as gene therapy vectors because of their low immunogenicity, long-term expression, and minimal genomic integration. Multiple serotypes exist (AAV1 to AAV9), and clinically approved vectors include AAV1 (Glybera), AAV2 (Luxturna, Upstaza), AAV5 (Roctavian, Hemgenix), and AAV9 (Zolgensma) [139]. Glybera (alipogene tiparvovec), the first gene therapy approved by the European Medicines Agency in 2012 for lipoprotein lipase deficiency, was later withdrawn in 2017 after its marketing authorization was not renewed. This decision was primarily attributed to commercial challenges, including its high cost and limited clinical adoption, rather than safety or efficacy concerns [140].

AAV8 has an icosahedral capsid composed of three structural proteins, VP1, VP2, and VP3, present at an approximate 1:1:10 stoichiometric ratio, with distinct surface-loop variations that influence receptor binding and transduction efficiency. Unlike AAV2, which uses a heparan sulfate receptor, AAV8 interacts primarily with laminin receptors, which contributes to altered cellular entry and higher transduction efficiency [141,142,143].

AAV8 shows broad tissue tropism and high transduction efficiency, particularly in the liver and retina. For ocular applications, subretinal injection is preferred because it bypasses the inner limiting membrane and directly targets photoreceptors and RPE cells, enabling more efficient gene delivery than intravitreal or systemic routes, which face substantial anatomical and diffusion barriers [143].

The *RLBP1* gene encodes CRALBP, a retinoid-binding protein in the RPE and Müller glia that supports 11-*cis*-retinoid recycling in the visual cycle. Loss-of-function mutations in RLBP1 impair chromophore regeneration, leading to delayed dark adaptation and progressive photoreceptor degeneration in retinitis pigmentosa [144].

Gene replacement can restore CRALBP expression and improve visual cycle kinetics and retinal function. One such therapy, CPK850, is an AAV8-based vector delivering RLBP1 that is currently being evaluated by subretinal administration in a Phase 1/2 clinical trial for retinitis pigmentosa. The trial primarily assesses safety and tolerability while also examining early efficacy outcomes such as improvements in dark adaptation and visual function.

More than 30 clinical trials are ongoing across multiple IRDs such as retinitis pigmentosa and choroideremia, and several Phase 1 to 3 studies have shown improved visual acuity, although some trials failed to meet their endpoints and reported adverse effects [145]. Clinical evidence indicates long-term benefit (up to 5 years) in some trials, but safety concerns such as inflammation and retinal damage, along with variability in outcomes, remain key challenges. Emerging approaches such as mRNA-based and non-viral therapies remain in early clinical or preclinical stages and aim to improve safety, scalability, and cost-effectiveness relative to current viral-vector approaches.

Several reviews surveyed gene therapy and genome editing approaches for ocular diseases [145,146,147]. Most of these therapies remain in development, however, and major challenges arise from ocular delivery barriers, including the blood–retinal barrier (BRB), the limited intraocular space, and the need for localized administration. Repeated intraocular injections further increase the risk of inflammation and immune responses, which can compromise safety and efficacy. Together, these factors represent major bottlenecks for the clinical translation of ocular gene therapy.

Despite these advances, major technical challenges persist, including limited vector cargo capacity, suboptimal retinal penetration, and safety concerns such as immune responses and retinal toxicity. These limitations have prompted growing interest in alternative delivery platforms, particularly non-viral systems such as lipid and polymeric nanoparticles, as well as mRNA-based therapeutics, which offer improved safety, scalable production, and avoidance of genomic integration.

### 5.2. Small-Molecule Inhibitors of Cellular and Serum RBPs

Retinol-binding proteins (RBPs), particularly RBP4, belong to the lipocalin family of lipid transfer proteins and mediate non-vesicular transport of retinoids through a conserved β-barrel (calyx) domain that binds hydrophobic ligands such as retinol. This architecture enables precise regulation of retinoid distribution and links RBPs to key processes such as metabolism, immune modulation, and cellular differentiation. Dysregulation of RBPs is implicated in metabolic, inflammatory, and retinal disorders, making them attractive therapeutic targets. Consequently, small-molecule inhibitors of RBP4 have been developed to disrupt retinol transport and modulate retinoid signaling, with potential in treating conditions such as insulin resistance, retinal degeneration, and certain cancers [148].

Unlike most retinoid-binding proteins, which act intracellularly, RBP4 is an extracellular serum protein [37]. Because extracellular targets are generally more readily accessible to therapeutics, RBP4 has been the main focus of drug development within the retinoid-binding protein family.

RBP4 inhibitors include both retinoid and non-retinoid scaffolds. The cellular inhibitor abn-CBD was described above, and this section focuses on inhibitors of serum RBP4. Fenretinide (N-(4-hydroxyphenyl)retinamide) is a retinol analogue that selectively inhibits RBP4 by preserving key hydrophobic interactions within the lipocalin pocket while replacing the isoprenoid tail with a phenylamide group [149]. Structural and crystallographic studies confirm minimal disruption of ligand–protein complementarity, supporting high-affinity binding. Fenretinide competitively displaces retinol with nanomolar potency (IC_50_ ≈ 56 nM), thereby modulating systemic retinoid transport [150]. Because bisretinoid lipofuscin such as A2E forms through non-enzymatic condensation of all-*trans*-retinal with phosphatidylethanolamine, reducing retinol flux into the retina is a key strategy to slow age-related macular degeneration (AMD) by limiting lipofuscin accumulation and the associated retinal toxicity.

Retinoid binding to RBP4 follows a “hand-in-glove” model, in which the trimethyl cyclohexene moiety anchors within the hydrophobic lipocalin core, conferring high affinity and specificity. The polyene chain further stabilizes the ligand–protein interaction, and its optimal length is critical, since shortening markedly reduces binding affinity. These observations indicate that both the hydrophobic core interactions and the integrity of the polyene chain govern RBP4–retinoid binding [148,151].

Non-retinoid RBP4 inhibitors such as A1120 were developed to avoid retinoid-associated toxicity while retaining high affinity (*K*i ~8.3 nM) through hydrogen bonds with Leu37, Tyr90, Gln98, and Arg121 and through hydrophobic contacts within the lipocalin domain [30]. A1120 showed poor liver microsomal stability, however, which prompted structural optimization. Derivatives such as BPN-14136 achieved improved stability, stronger binding (IC_50_ ~12.8 nM), and greater efficacy, reducing circulating RBP4 by up to 90% in retinal disease models [152].

These inhibitors are particularly relevant to retinal disorders such as Stargardt disease and AMD, in which lowering RBP4 reduces retinol delivery to the retina, and thereby limits the accumulation of toxic bisretinoid lipofuscin. Advanced candidates such as tinlarebant and STG-001 are currently in Phase III trials, underscoring the clinical potential of non-retinoid RBP4 inhibition, although challenges such as transthyretin (TTR) destabilization and amyloidosis risk remain [148].

Non-retinoid CRBP1 inhibitors such as abn-CBD instead target intracellular retinol-binding, forming key hydrogen bonds with Ala33, Lys40, and Gln128, achieving high affinity (*K*i ~67 nM) and protecting against light-induced retinal damage in vivo. Newer scaffolds (inhibitors 1–6) further explore CRBP1 targeting, although with lower affinity and possible selectivity limitations arising from the conserved binding residues [32]. CRBP1 inhibition is a promising strategy to modulate intracellular retinol metabolism in RPE while potentially avoiding the systemic retinol depletion associated with RBP4 inhibitors. However, selectivity, metabolic stability, and long-term safety must be addressed before these inhibitors can be translated into clinical use for retinal diseases. Table 2 summarizes all small-molecule inhibitors that target retinoid-binding proteins for retinal disease.

## 6. Challenges and Future Perspectives

The intracellular lipid-binding protein (iLBP) family comprises structurally related retinoid-binding proteins that share conserved ligand-binding features while participating in distinct biological processes. A major challenge in targeting this family is the high similarity of their ligand-binding pockets. Because CRBP, CRABP, and related proteins share conserved hydrophobic cavities for retinoid binding, a compound designed for one protein may also bind others, causing off-target effects and making achieving subtype selectivity difficult. Future work focusing on subtle differences in pocket flexibility, conformational dynamics, and allosteric regulation may enable the design of more selective modulators.

Although abn-CBD and the newer scaffolds show that CRBP1 can be pharmacologically targeted, the available in vivo evidence remains very limited. To date, these compounds have mainly been evaluated in biochemical assays, with limited in vivo testing in a light-induced retinal damage model. Their long-term safety, tissue distribution, and therapeutic efficacy remain to be established. Evaluation in clinically relevant disease models will therefore be essential to determine the translational potential of CRBP1-targeted therapies.

At present, no small-molecule drug targeting CRBP, CRALBP, or CRABP has advanced to clinical development. Gene-based therapeutic approaches, however, have shown encouraging progress. In particular, CPK850, an AAV8-based gene replacement therapy delivering the *RLBP1* gene, is currently under Phase 1/2 evaluation for CRALBP deficiency.

Despite strong links of retinoid-binding proteins to retinal disease, retinoid signaling, neurobiology, and cancer, their therapeutic translation remains at an early stage. A major limitation is that these proteins serve essential physiological roles in retinoid homeostasis, which makes selective modulation difficult without disrupting normal cellular function. Even so, CRABP1 has emerged as a potential neuroprotective target through its role in retinoic acid signaling and cellular stress responses, whereas CRABP2 is increasingly studied as both a cancer biomarker and a therapeutic target linked to tumor progression and retinoid sensitivity.

Future therapeutic development is likely to benefit from advances in structural biology, artificial intelligence-assisted drug discovery, and chemoproteomics. Cryo-electron microscopy (cryo-EM) may allow visualization of dynamic conformational states that are difficult to capture by conventional crystallography, particularly for retinoid-binding proteins acting in the membrane-proximal environment of the visual cycle. In parallel, machine learning-guided virtual screening, ensemble docking, and generative molecular design could expand the chemical diversity of candidate modulators beyond conventional high-throughput screening libraries. Chemoproteomic platforms, including activity-based protein profiling (ABPP), may further identify endogenous ligands and novel allosteric sites within these proteins. Induced-proximity technologies such as PROTACs and molecular glues may also enable tissue-selective degradation of disease-associated targets in the RPE.

Overall, although substantial pharmacological and translational challenges remain, the iLBP family represents a promising and still underexplored therapeutic target class for retinal diseases, neurodegenerative disorders, and retinoid-responsive cancers. Continued integration of structural, biochemical, pharmacological, and translational approaches will be essential to advance next-generation iLBP-targeted therapeutics.

## 7. Conclusions

Among the intracellular retinoid-binding proteins reviewed here, CRALBP is the most clinically validated target owing to its direct genetic link to inherited retinal dystrophies and the ongoing AAV8-based gene therapy trial (CPK850). Even so, small-molecule modulators targeting its allosteric lid mechanism remain unexplored.

CRABP isoforms are currently among the most promising therapeutic targets in the iLBP family, given their potential roles in neuroprotection, tumor progression, and retinoid sensitivity. Their development is still constrained by limited structural characterization of isoform-selective ligands, the scarcity of robust in vivo pharmacological data beyond genetic models, incomplete selectivity profiling across the iLBP family, and the absence of tissue-targeted delivery methods. CRBP1 currently has the strongest small-molecule pharmacological evidence, supported by high-resolution co-crystal structures, a defined three-component pharmacophore, and in vivo findings that CRBP1 inactivation reduces bisretinoid accumulation and protects against light-induced retinal degeneration. Further structural, pharmacokinetic, and disease-model studies will nonetheless be needed to optimize first-generation compounds and advance them toward clinically viable therapeutics.

## Figures and Tables

**Table 1:** Comparative analysis of CRBP, CRABP, and CRALBP.

Isoform (Gene)	Ligand Class	Primary Role	Key Tissues/ Cells	Core Partners	Binding Affinity (*K*d)	Key Binding Residues	Ref
CRBP1 (*RBP1*)	all-*trans*- retinol/ retinal	Retinol transport, storage, and delivery to LRAT/RDH	RPE, liver (HSCs, hepatocytes), kidney, lung	LRAT, RDH1/10RALDH1/2	all-*trans*-retinol (3 ± 2 nM)	(Lys40, Gln108)	[45]
CRBP2 (*RBP2*)	all-*trans* retinol/retinal, monoacylglycerols	Dietary vitamin A absorption	Small intestine (enterocytes)	LRAT	all-*trans*-retinal (10 to 50 nM)	Thr51, Val62	[54]
CRBP3 (*RBP7*)	all-*trans*-retinol	Adipogenesis and lipid metabolism. Direct transcriptional target of PPARγ; induced during adipocyte differentiation	Mature adipocytes (WAT & BAT), breast epithelium	PPARγ	(60 nM)	(Gln108–His108 switch)	[94,95]
CRBP4 (*RBP5*)	all-*trans*- retinol/9-*cis*/13-*cis*- retinol	Hepatic/renal retinoid metabolism	Liver, kidney	Unknown	(~200 nM)	(Gln108–His108 switch)	[64,96]
CRALBP (*RLBP1*)	11-*cis*-retinal/11-*cis*-retinol	Chaperone for 11-*cis*-retinoids in visual cycle; accelerates RPE65-mediated isomerization	RPE, Müller glial cells	RPE65, RDH5, retinol isomerase	11-*cis*-retinal (15 to 21 nM) 11-*cis*-retinol (~30 nM)	(Tyr179, Gln210, Lys221)	[70,73,97]
CRABP1 (*CRABP1*)	all-*trans*-retinoic acid (high affinity) also binds 9-*cis* and 13-*cis*-RA	Regulates retinoic acid bioavailability via sequestration	Broad (brain, testis, limb mesenchyme)	CYP26A1/B1, CYP26C1	(0.1 to 1 nM) apo-CRABP-I *K*_i_ for CYP26A1 (0.39 nM)	(Arg112, Arg132 and Tyr134)	[98,99]
CRABP2 (*CRABP2*)	all-*trans*- retinoic acid	Nuclear retinoic acid delivery, translocates with RA to RAR;	Epidermis, squamous epithelia, uterus	RAR/RXR	(2 to 14 nM)	(Arg112, Arg133 and Tyr135)	[100]

The nomenclature of the CRBP family follows Folli et al. [35] and Vogel et al. [101]. Data for CRBP4 refer to the mouse ortholog. Gln108 is the native retinol-binding residue, whereas Gln128 interacts with the inhibitor in the co-crystal structure.

**Table 2:** Small-molecule inhibitors targeting retinoid-binding proteins CRBP1 and RBP for retinal disease.

Compound	Binding Affinity	Target	Metric	Mechanism	Therapeutic Condition	Clinical Stage	Ref.
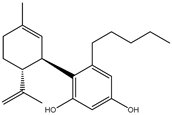 abn-CBD	67 nM	CRBP1	*K*i	Competitive inhibitor, blocks intracellular retinol binding	Retinal degeneration (light-induced)	Preclinical	[31]
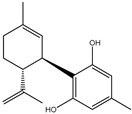 CBDO	1.78 µM	CRBP1	*K*i	Competitive inhibitor, abn-CBD analogue with reversed ortho/para-hydroxyl groups that blocks retinol binding	Retinal degeneration	Preclinical	[31]
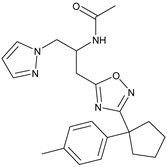 Non-retinoid scaffold	7.1 µM	CRBP1	*K*i	Competitive CRBP1 inhibitor, oxadiazole scaffold occupying the retinol-binding pocket	Retinal degeneration	Preclinical	[31]
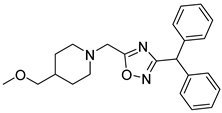 Non-retinoid scaffold	8.3 µM	CRBP1	*K*i	Competitive CRBP1 inhibitor, oxadiazole scaffold occupying the retinol-binding pocket	Retinal degeneration	Preclinical	[31]
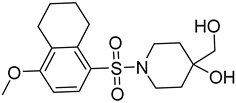 Non-retinoid scaffold	9.5 µM	CRBP1	*K*i	Competitive CRBP1 inhibitor, sulfonyl-containing scaffold occupying the retinol-binding pocket	Retinal degeneration	Preclinical	[31]
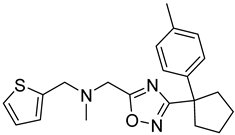 Non-retinoid scaffold	9.0 µM	CRBP1	*K*i	Competitive CRBP1 inhibitor, oxadiazole scaffold occupying the retinol-binding pocket	Retinal degeneration	Preclinical	[31]
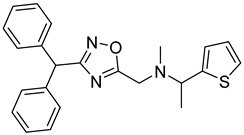 Non-retinoid scaffold	10.6 µM	CRBP1	*K*i	Competitive CRBP1 inhibitor, oxadiazole scaffold occupying the retinol-binding pocket	Retinal degeneration	Preclinical	[31]
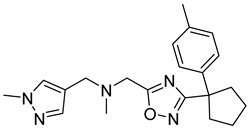 Non-retinoid scaffold	10.7 µM	CRBP1	*K*i	Competitive CRBP1 inhibitor, oxadiazole scaffold occupying the retinol-binding pocket	Retinal degeneration	Preclinical	[31]
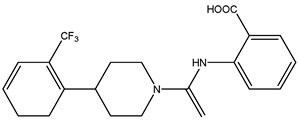 A1120	8.3 nM	RBP4	*K*i	Competitive RBP4 antagonist that reduces retinol delivery to the retina and bisretinoid formation	AMD and Stargardt disease	Preclinical	[30,150]
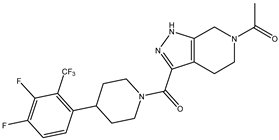 Tinlarebant	NR *	RBP4	-	RBP4-TTR complex disruption, with reduced retinal bisretinoid accumulation	Stargardt disease (adolescent), AMD	Phase III (DRAGON trial)	[153]
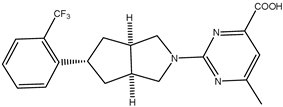 BPN-14136	12.8 nM	RBP4	IC_50_	Improved RBP4 inhibition and stability	AMD and Stargardt disease	Preclinical	[152]
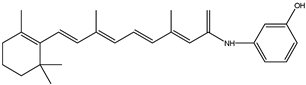 Fenretinide	56 nM	RBP4	IC_50_	Retinol displacement from RBP4	Geographic atrophy, AMD	Phase II (completed)	[150]

* NR = Not Reported.

## Data Availability

No new data were created or analyzed in this study. Data sharing is not applicable to this article.

## Abbreviations

AMD	Age-related macular degeneration
CRBP1	Cellular retinol-binding protein 1
RBP4	Retinol-binding protein 4
CRBP2	Cellular retinol-binding protein 2
CRBP3	Cellular retinol-binding protein 3
CRBP4	Cellular retinol-binding protein 4
atROL	All-*trans*-retinol
abn-CBD	Abnormal cannabidiol
CRALBP	Cellular retinaldehyde-binding protein
RPE	Retinal pigment epithelium
HTS	High-throughput screening
abn-CBDO	(5-methyl-4-[(1*R*,6*R*)-3-methyl-6-prop-1-en-2-ylcyclohex-2-en-1-yl]benzene-1,3-diol)
CRABP	Cellular retinoic acid-binding protein
LRAT	Lecithin: retinol acyltransferase
SAR	Structure–activity relationship
cryo-EM	Cryo-electron microscopy
iLBP	Intracellular lipid-binding protein
